# Fire Design Equation for Steel–Polymer Composite Floors in Thermal Fields Via Finite Element Analysis

**DOI:** 10.3390/ma13235573

**Published:** 2020-12-07

**Authors:** Min Jae Park, Jaehoon Bae, Jaeho Ryu, Young K. Ju

**Affiliations:** 1 School of Civil, Environmental, and Architectural Engineering, Korea University, Seoul 02481, Korea; alswo8739@korea.ac.kr (M.J.P.); skycity-bjh@korea.ac.kr (J.B.); 2 Technical Research Center, TechSquare Co., Ltd., Seoul 06710, Korea; jryu@techsq.co.kr

**Keywords:** steel–polymer composite floor, insulation performance, finite element analysis, full-scale fire test, fire resistance, thermal field

## Abstract

Owing to the development of new materials that enhance structural members in the construction field, steel–polymer composite floors have been developed and applied to steel structures. Similar to a sandwich system, steel–polymer composite floors consist of polymers between two steel plates. The structural performance of full-scale composite floors at ambient conditions has been investigated. Additionally, experiments were conducted on analytical models to predict both thermal behavior under fire, including fire resistance based on a small-scale furnace. To evaluate the fire resistance of full-scale steel–polymer composite floors, the thermal behavior and temperature distribution of composite floors should be investigated. Therefore, the temperature distributions of the full-scale composite floors were estimated using the verified analytical model in this study. Furthermore, to determine the fire design equation of steel–polymer composite floors in the thermal field, the correlations between variables were investigated, such as the thickness of top and bottom steel plates and polymers, as well as the fire resistance in the thermal field.

## 1. Introduction

**Figure 1:**
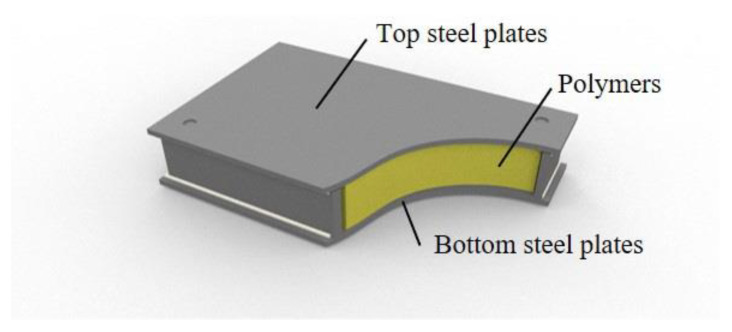
Steel–polymer composite floors.

For centuries, only a few materials, such as steel, concrete, and timber, have been used in structural engineering. However, in recent years, new structural materials have been developed to improve the original structural members that consist of steel and reinforced concrete. First, glass fiber-reinforced plastic (GFRP) was developed to strengthen reinforced concrete [1,2,3]. Saadatmanesh et al. conducted four-point bending tests to evaluate the flexural strength of reinforced concrete beams strengthened with GFRP plates [1]. An et al. presented analytical models to predict the stresses and deformations in concrete beams strengthened with fiber-reinforced plastic plates [2]. Ryu et al. conducted tests and finite element analyses to evaluate the shear resistance of a new composite floor system containing GFRP [3]. A brake-friction pad composed of rubber was applied to a hybrid damper with a steel-strip damper designed to resist earthquakes [4,5]. Carbon fiber and polymers have also been investigated to strengthen the structural performance of reinforced concrete [6,7,8,9]. Lee et al. investigated cement-based sensors with hybrid conductive fillers [6], and Yoo and Yoon estimated the impact resistance of steel fiber-reinforced concrete slabs strengthened with fiber-reinforced polymer sheets [7]. Dunaj et al. proposed a modeling method to assess the dynamic properties of steel–polymer concrete frames [8]. Heidarnezhad et al. studied the mechanical properties of lightweight polymer concrete depending on polymer content and temperature [9]. In addition, steel–polymer composite floors have been developed and applied to steel structures instead of steel–concrete composite floors [10]. The steel–polymer composite floor is a type of sandwich system in which core materials are located between skin materials. Polymers in the composite floors were placed between the top and bottom steel plates, as shown in Figure 1.

Steel–polymer composite floors are prefabricated systems that can reduce the duration of construction owing to dry construction methods such as welded and bolted installations. The thickness of composite floors typically ranges from 25 mm to 80 mm, whereas the general floor system in steel structures has a thickness of 100–200 mm. Since the bond between polymers and steel exhibits a strength capacity that is usually 10 times larger than that between concrete and steel, the section of composite floors can sustain the section under large deformations with shallow thickness [10]. Owing to this feature, polymers, which are elastomers that have large failure strains on the composite floors, were targeted to exhibit strong bond strengths in the early development stage. To investigate the fire resistance performance of composite floors, fire-resistant polymers have been developed, thus enhancing fire resistance [11]. However, conducting fire tests based on Korean Standards (KS) was very difficult in Korea [12,13,14]. There are only a few authorized furnaces in Korea, and they require a lot of time and are expensive. In addition, according to the traffic rules, it is impractical to move specimens with a minimum size of 3.7 m × 3.0 m on major roads. These limitations indicate that efficient studies requiring lower costs and less time are needed, such as small-scale tests or analytical studies. Consequently, using finite element methods and small-scale furnace tests, analytical studies were conducted to investigate the fire behavior and fire resistance of steel–polymer composite floors [11,15]. The performance of fire resistance was evaluated using small-scale furnace tests and alternative fire-severity methods [15]. A thermal-contact, conductance-based thermal behavior analytical model for the composite floors was developed and verified using furnace test results [11]. The temperature distribution of the composite floors at elevated temperatures is a prerequisite that determines mechanical properties depending on the temperature. The more errors that are generated in the thermal field, the larger the appearance of uncertainties that cannot be investigated when predicting structural behavior; this includes both those created in terms of the thermal field and those created in terms of the structural field. Therefore, studies on the thermal field should be prioritized. However, generalized results for the fire design of composite floors in the thermal field have not been investigated. Therefore, in this study, the fire design in the thermal field (known as the insulation performance) for steel–polymer composite floors was proposed using the results obtained by finite element analysis with ABAQUS/CAE 2017. The validation of the finite element model that had already been obtained from various-scale fire tests was achieved again by comparing it with full-scale fire tests based on the KS.

## 2. Verification of Analysis Model

### 2.1. Thermal Behavior Analytical Model

**Figure 2:**
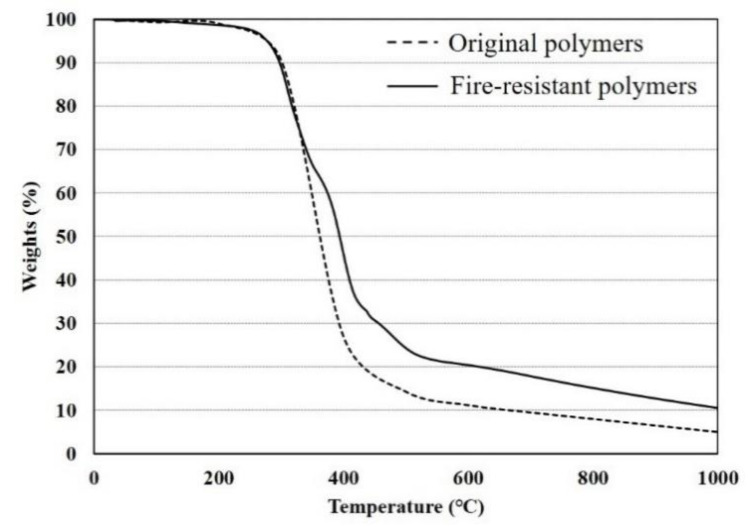
TGA results of original and fire-resistant polymers [11].

The thermal behavior analytical model for steel–polymer composite floors focuses on thermal contact conductance, which is the contact issue between polymers and steel. Thermal contact conductance is defined by an invisible gap and contact surfaces. If there are more contact surfaces, the thermal contact conductance would be higher. This means that more heat flux can be transferred between the two materials. The contact surfaces between two materials are related to physical factors, such that the thermal contact conductance at elevated temperatures can be altered by physical properties such as residual mass. The residual mass at elevated temperatures can be obtained from thermal gravimetric analysis (TGA) obtained from previous study [11], as shown in Figure 2. The reduction ratio of thermal contact conductance at elevated temperatures agrees with the pattern of the TGA results, and the value of the thermal contact conductance at ambient temperatures is 250 W/m^2^·K.

**Figure 3:**
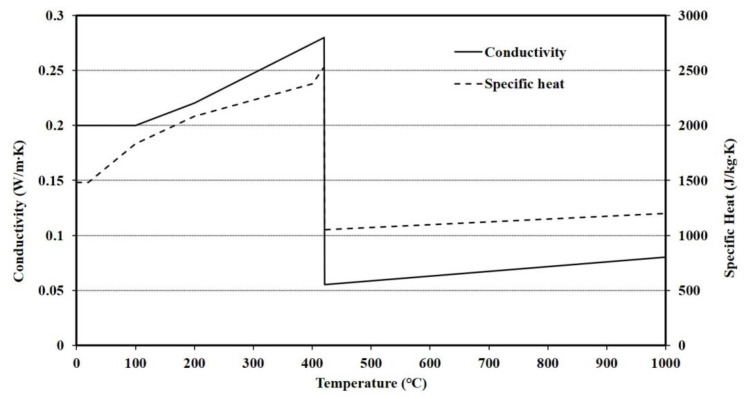
Thermal properties of polymers at elevated temperatures [11].

The thermal properties of steel at elevated temperatures were obtained from Eurocode 3 [16], and the values of the polymers at elevated temperatures obtained from previous study [11] are shown in Figure 3.

### 2.2. Full-Scale Fire Tests

**Figure 4:**
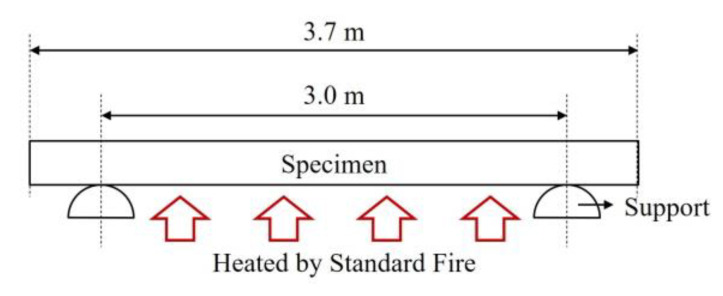
Floor specifications for fire tests based on previous version (2014) of Korean Standards [17,18].

Full-scale fire tests are required to follow Korean Standards, and their results are used to validate the analysis model for full-scale specimens. Because this fire test was conducted before the publication of new standards (2019) [12,13], it followed the standards of the previous version (2014) [17,18]. According to the standards, the lengths of the heated support and specimen are 3 m and 3.7 m, respectively, whereas the width of the specimen is 3 m, as shown in Figure 4.

**Figure 5:**
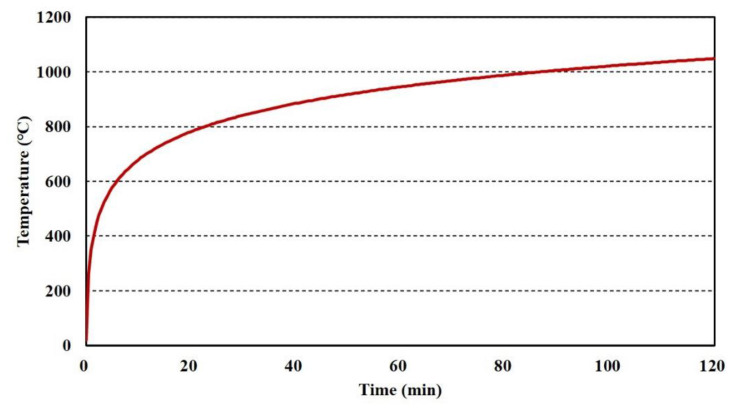
Standard fire curve for fire tests based on Korean Standards [12,13,17,18].

For the floor, the lower parts of the specimen were heated by a standard fire curve during the target time, as illustrated in Figure 5. The thicknesses of the top and bottom steel plates and polymers were 3, 3, and 35 mm, respectively. The width of the parameter steel bar was 40 mm. The temperature at the center point of the specimen was measured, and the result was compared with that obtained from the analysis to validate the proposed model.

### 2.3. Validation for Full-Scale Fire Tests

**Figure 6:**
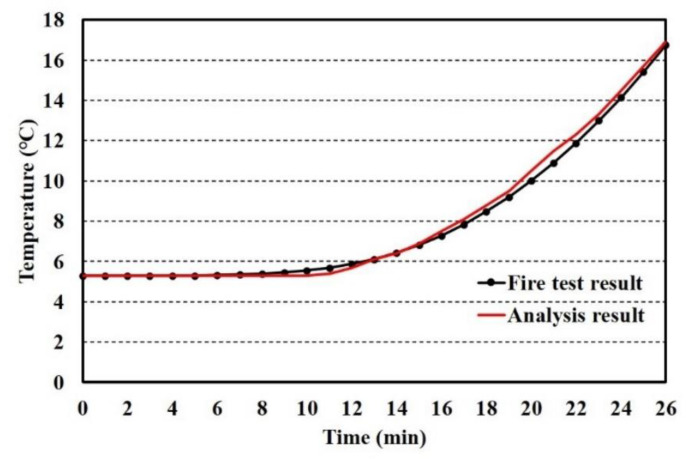
Comparison between fire test and analysis results.

The full-scale fire test was terminated after 26 min owing to the unexpected failure of the weld between the parameter bar and top steel plates. The time history of temperature at the center point of the upper surface was obtained, and the results are shown in Figure 6, together with the analysis result. ABAQUS/CAE 2017 was utilized for conducting the finite element analysis. DC3D8, an eight-node linear heat transfer brick, was selected, with a mesh size of 0.1 m. When the temperature of the test reached 16.9 °C, the temperature of analysis was at 16.7 °C. The error of the thermal behavior analytical model for a full-scale fire test is less than 1%. Thus, it is reasonable to adapt the thermal behavior analytical model for predicting the temperature of full-scale steel–polymer composite floors.

## 3. Full-Scale Analysis Model for Fire Design

### 3.1. Modeling for Full-Scale Test

**Figure 7:**
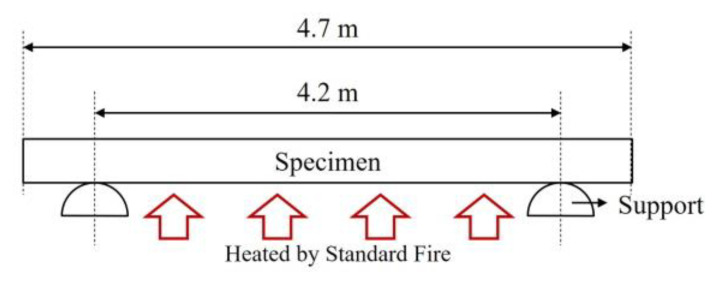
Floor specification for fire tests based on revised version (2019) of Korean Standards [12,13].

Revised contents of full-scale fire tests were extended based on the standards, lengths of heated support, and specimen (Figure 7). However, the width of the specimen remained constant at 3.0 m. Therefore, the full-scale dimension of specimens for the fire tests would be 4.7 m × 3.0 m.

**Figure 8:**
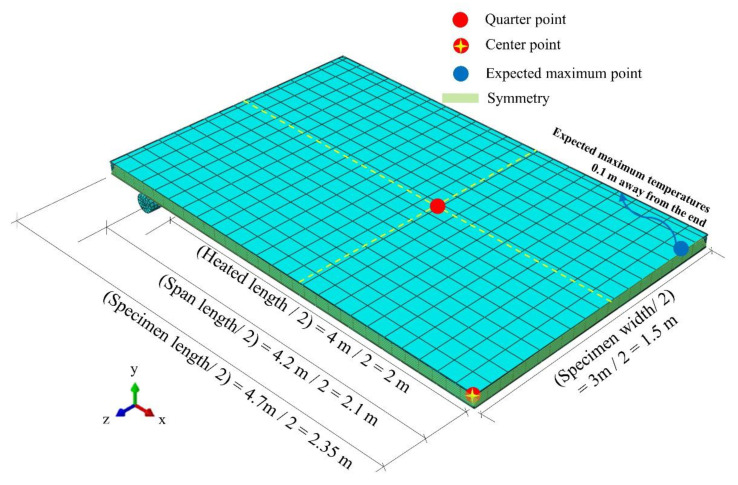
Configuration of full-scale fire tests.

To evaluate the fire resistance in the thermal field, temperatures of the upper surface should be measured and evaluated using established criteria. Effectively, five points located at a center point and four quarter points should be measured. Additionally, the expected maximum temperature point should be measured. If the point is located at the end of the specimen, the point should be located 0.1 m from the end. Figure 8 presents the descriptions of the full-scale test with a quarter model that can reduce the computational cost of the analysis with x- and z-axis symmetries.

Similar to the validation model, the thermal properties of steel were obtained from Eurocode 3 [16], whereas the thermal properties of the polymers were obtained from a previous study [11]. Based on the thermal-contact conductance-based thermal analytical model, the interfacial properties between polymers and steel at elevated temperatures can be obtained from the TGA results at 250 W/m^2^·K and ambient temperatures. The boundary conditions, coefficient of heat transfer by convection, surface emissivity of the specimen, and emissivity of the fire of exposed and unexposed surface to standard fire, were determined as per the recommendations by Eurocode 1 [19]. Since materials of exposed and unexposed surfaces are made of steel, their coefficients of heat transfer by convection are 25 W/m^2^·K and 4 W/m^2^·K, respectively, whereas the surface emissivity of the specimen is 0.7. For the standard fire, its emissivity is set as 1.0. In addition, DC3D8, an eight-node linear heat transfer brick, was selected, with a mesh size of 0.1 m that was an appropriate size to conduct heat transfer analysis for the composite floor, according to previous study [11].

### 3.2. Variables of Analysis Models

**Figure 9:**
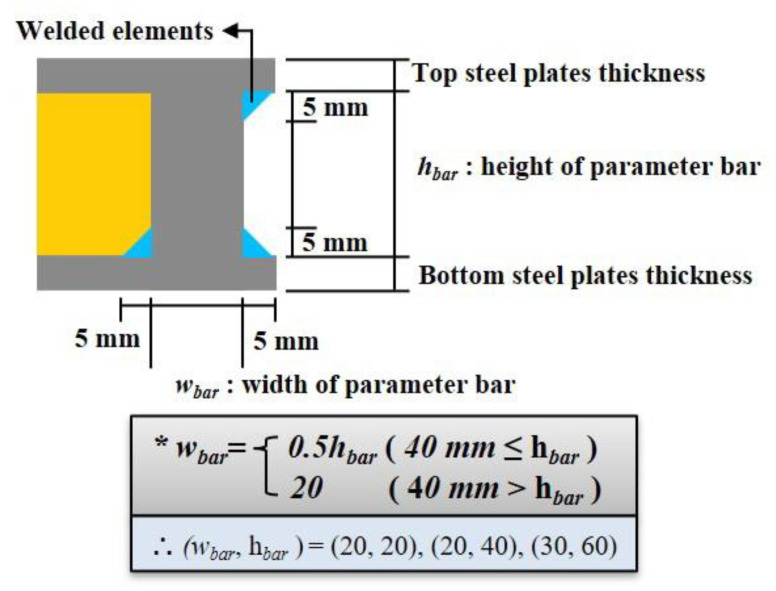
Detailed description of parameter bars.

**Figure 10:**
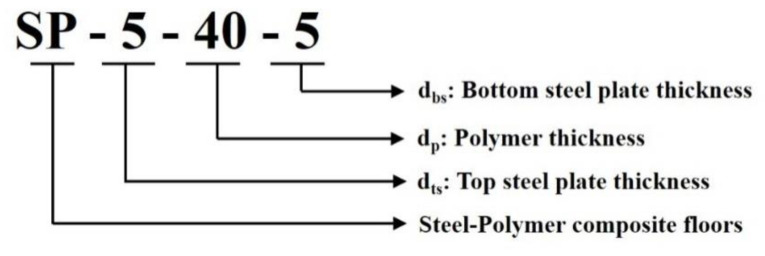
Description of analysis model label.

The main variables of the analysis models were the thickness of the top (*d_ts_*) and bottom (*d_bs_*) steel plates and polymers (*d_p_*). The three groups of specimens are classified according to d_p_: 20, 40, and 60 mm. The thicknesses of the top (*d_ts_*) and bottom (*d_bs_*) steel plates ranged from 5 mm to 20 mm. In addition, the parameter bars, the other variables that surround the polymer between the top and bottom steel plates, were considered. For the parameter bar, its width (*w_bar_*) represents half of its height (*h_bar_*). However, there is a limitation to the minimum size of the parameter bar in connecting the other elements and installing the connection with beams. To address this connection issue, the height of the parameter bar should exceed 20 mm. The welded elements with parameter bars, as well as top and bottom steel plates, were modeled because the welded elements altered the heat flow regardless of whether they were considered or not. The size of the welded elements was obtained from previous studies [11,15], as shown in Figure 9. Figure 10 describes the implication of the analysis model label.

## 4. Fire Design Equation in Thermal Field

### 4.1. Analysis Results

Regarding floor members, there are two criteria for fire resistance in the thermal field. These criteria are represented in terms of change in temperature (Δ*T*), as shown in Equation (1). One criterion is the maximum temperature change, while the other is the average temperature change on the unexposed surface. Since the six points including the expected maximum temperature were measured, the maximum and average temperature changes can be expressed in Equations (2) and (3).

(1) ΔT=T(t)−T(0) 

(2) $$\Delta T_{max}=\;Max\left[\Delta T_{c},\Delta T_{q},\Delta T_{e}\right]$$

(3) $$\Delta T_{average}=\;\frac{\Delta T_{c}+\Delta 4T_{q}+\Delta T_{e}}{6}$$

where Δ*T_max_*, Δ*T_c_*, Δ*T_q_*, Δ*T_e_*, and Δ*T_average_* represent the maximum change in temperatures of the unexposed surface, change in temperature of the center point, change in temperatures of the quarter points, change in temperatures of the expected maximum temperature point, and average change in temperatures of the unexposed surface, respectively. According to the criteria of Korean Standards, Δ*T_max_* and Δ*T_average_* should not exceed 180 °C and 140 °C, respectively.

**Figure 11:**
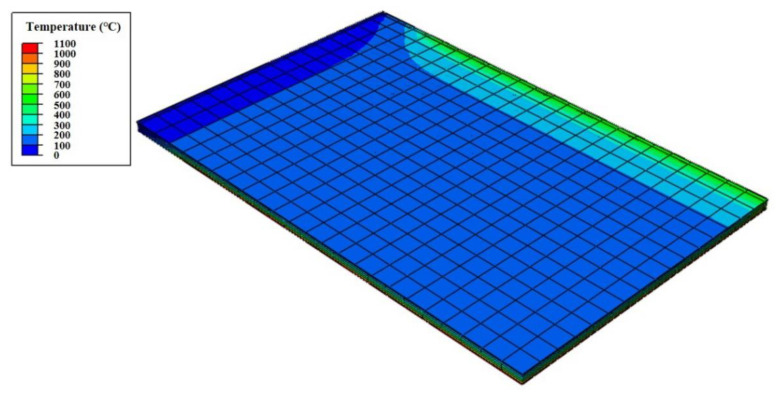
Finite element analysis results (SP-5-40-5).

**Figure 12:**
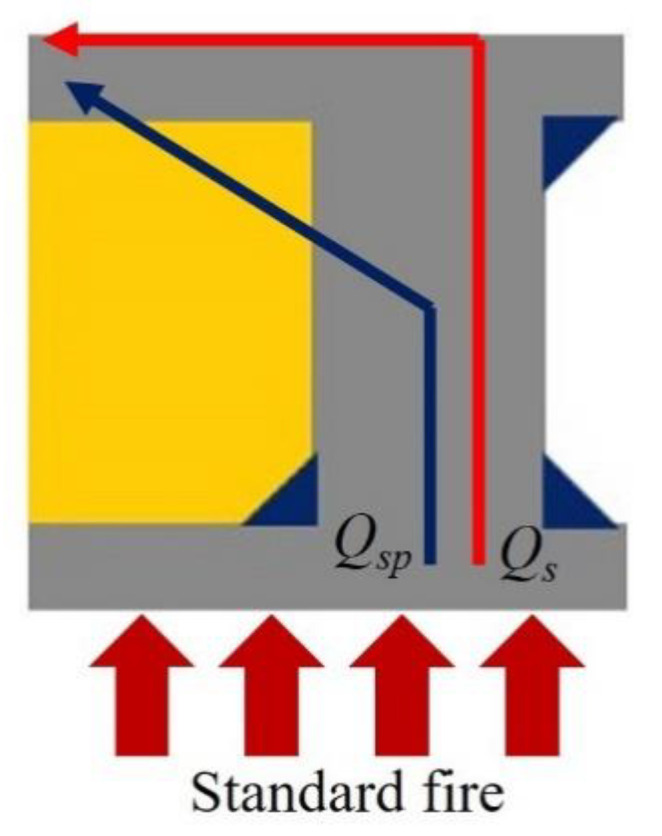
Heat flow path at the parameter bar.

**Figure 13:**
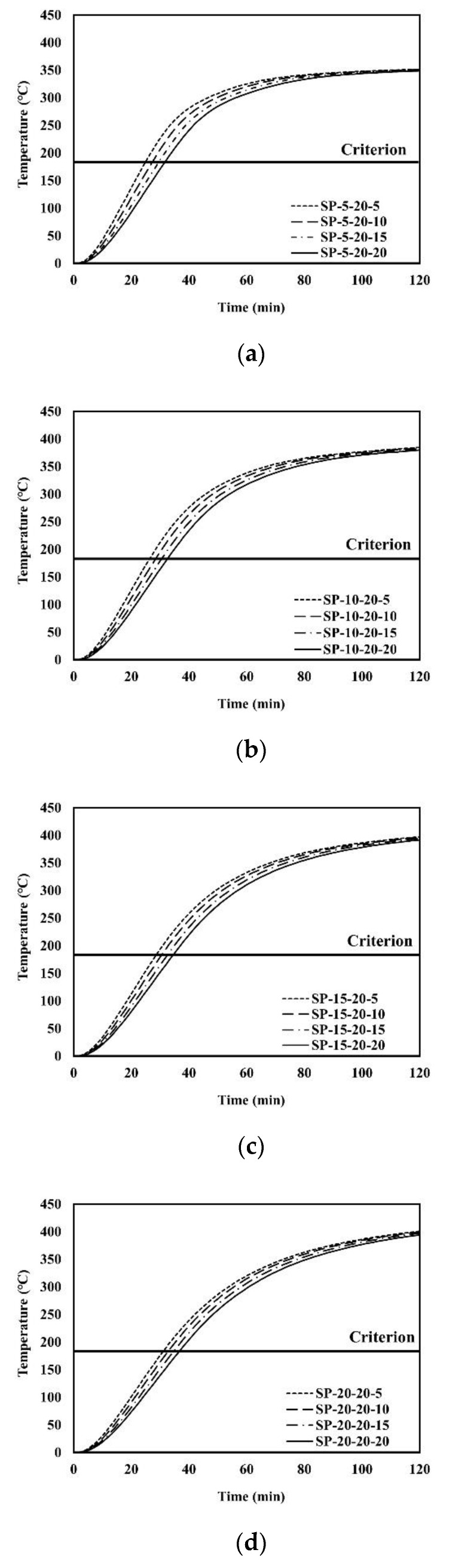
Time history of maximum temperatures with *d_p_* = 20 mm: (**a**) SP-5-20-x; (**b**) SP-10-20-x; (**c**) SP-15-20-x; (**d**) SP-20-20-x.

**Figure 14:**
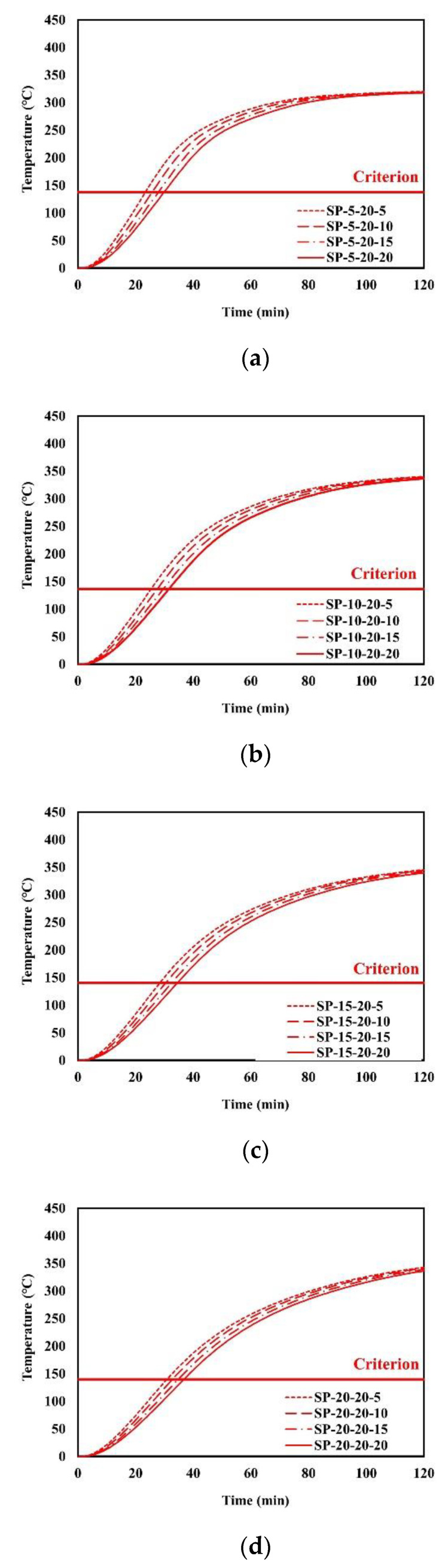
Time history of average temperatures with *d_p_* = 20 mm: (**a**) SP-5-20-x; (**b**) SP-10-20-x; (**c**) SP-15-20-x; (**d**) SP-20-20-x.

**Figure 15:**
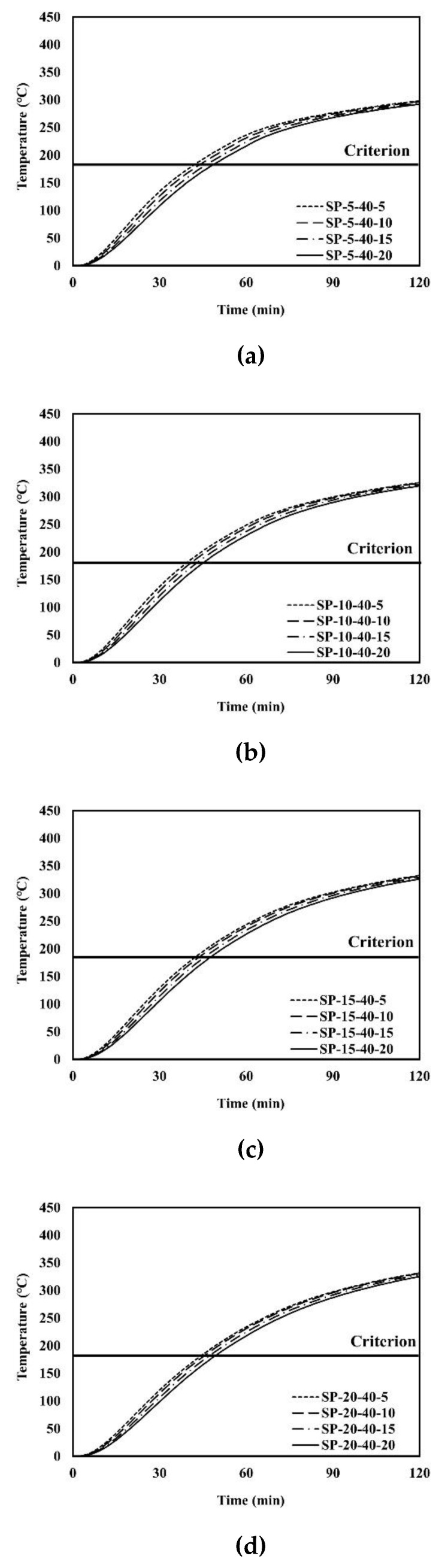
Time history of maximum temperatures with *dp* = 40 mm: (**a**) SP-5-40-x; (**b**) SP-40-20-x; (**c**) SP-15-40-x; (**d**) SP-20-40-x.

**Figure 16:**
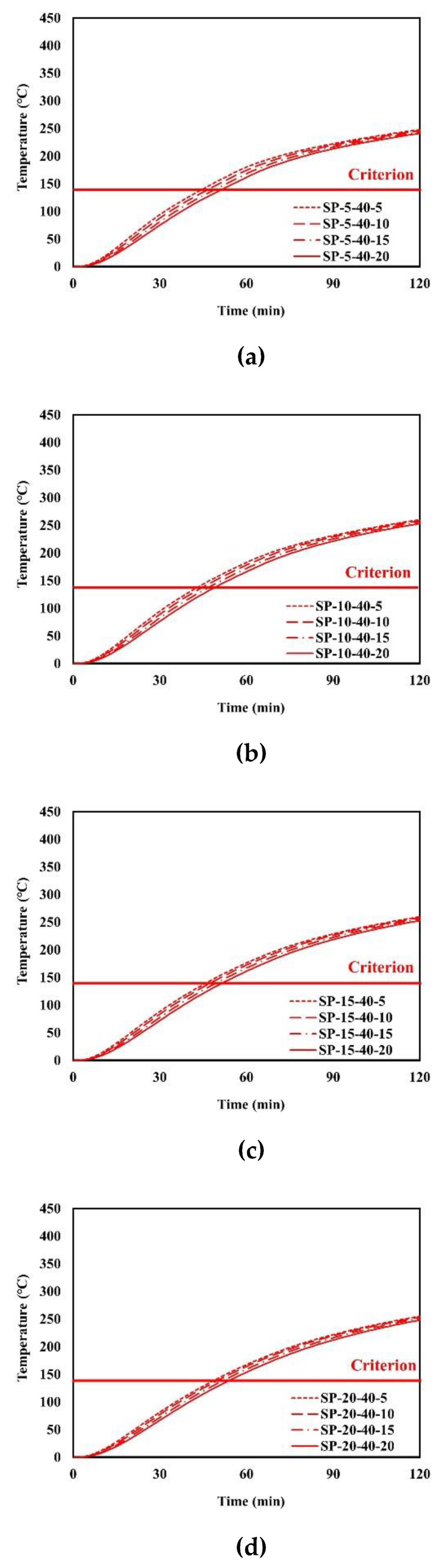
Time history of average temperatures with *dp* = 40 mm: (**a**) SP-5-40-x; (**b**) SP-10-40-x; (**c**) SP-15-40-x; (**d**) SP-20-40-x.

**Figure 17:**
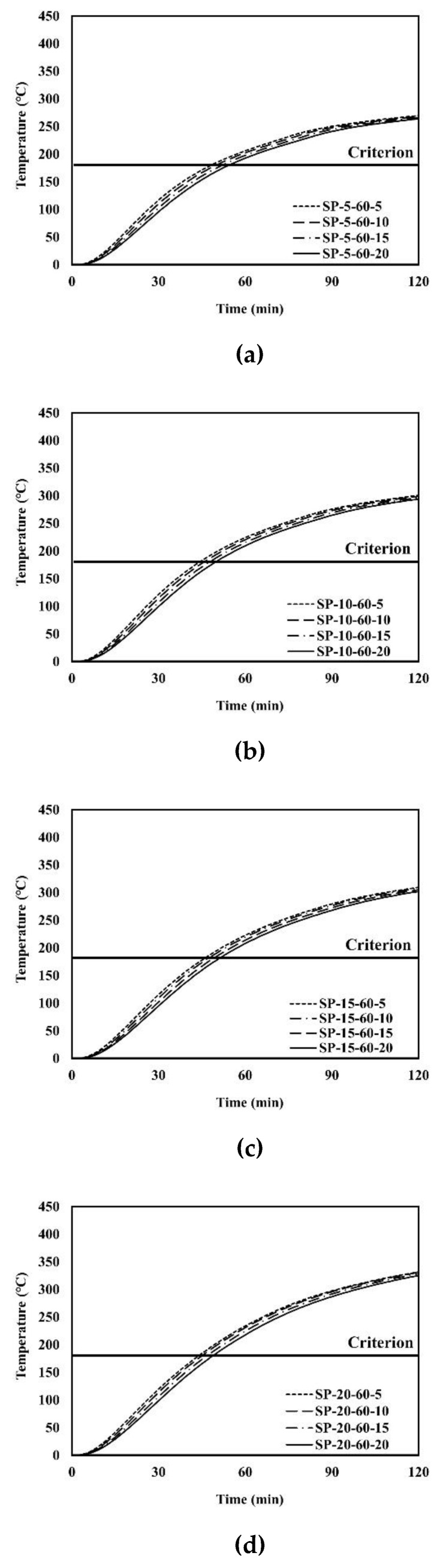
Time history of maximum temperatures with *dp* = 60 mm: (**a**) SP-5-60-x; (**b**) SP-60-20-x; (**c**) SP-15-60-x; (**d**) SP-20-60-x.

**Figure 18:**
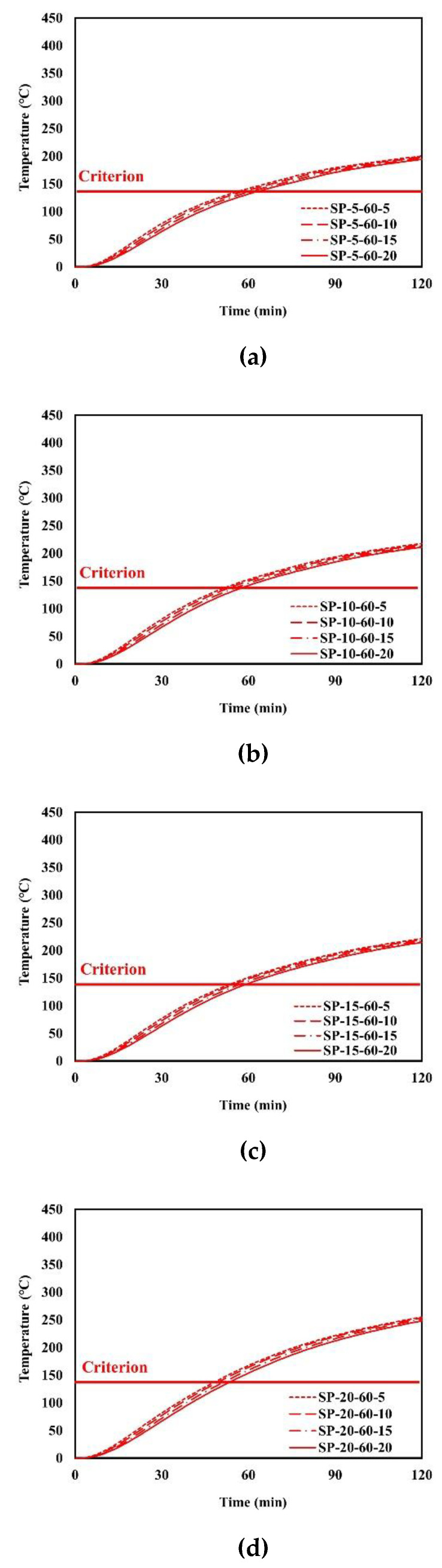
Time history of average temperatures with *dp* = 60 mm: (**a**) SP-5-60-x; (**b**) SP-10-60-x; (**c**) SP-15-60-x; (**d**) SP-20-60-x.

An example of the finite element analysis results is shown in Figure 11. Temperatures at the unexposed surface near the parameter bars were higher than those at other areas on the unexposed surface. The parameter bar guided the heat generated by the standard fire to approach the unexposed surface with ease. Because of the parameter bar, the temperature distribution of the composite floor exhibits an irregular pattern. In Figure 12, the heat energy (*Q_sp_*) of polymers and that of steel alone (*Q_s_*) create the temperature distribution and temperature alterations on the exposed surface. Figure 13, Figure 14, Figure 15, Figure 16, Figure 17 and Figure 18 show the time histories of temperature changes of the whole analysis results, with the criteria for maximum and average temperatures on the unexposed surface. The fire resistance performance of steel–polymer composite floors in the thermal field is determined as the time when the maximum or average changes in temperature exceed the criteria.

### 4.2. Fire Design Equation in Thermal Field

**Figure 19:**
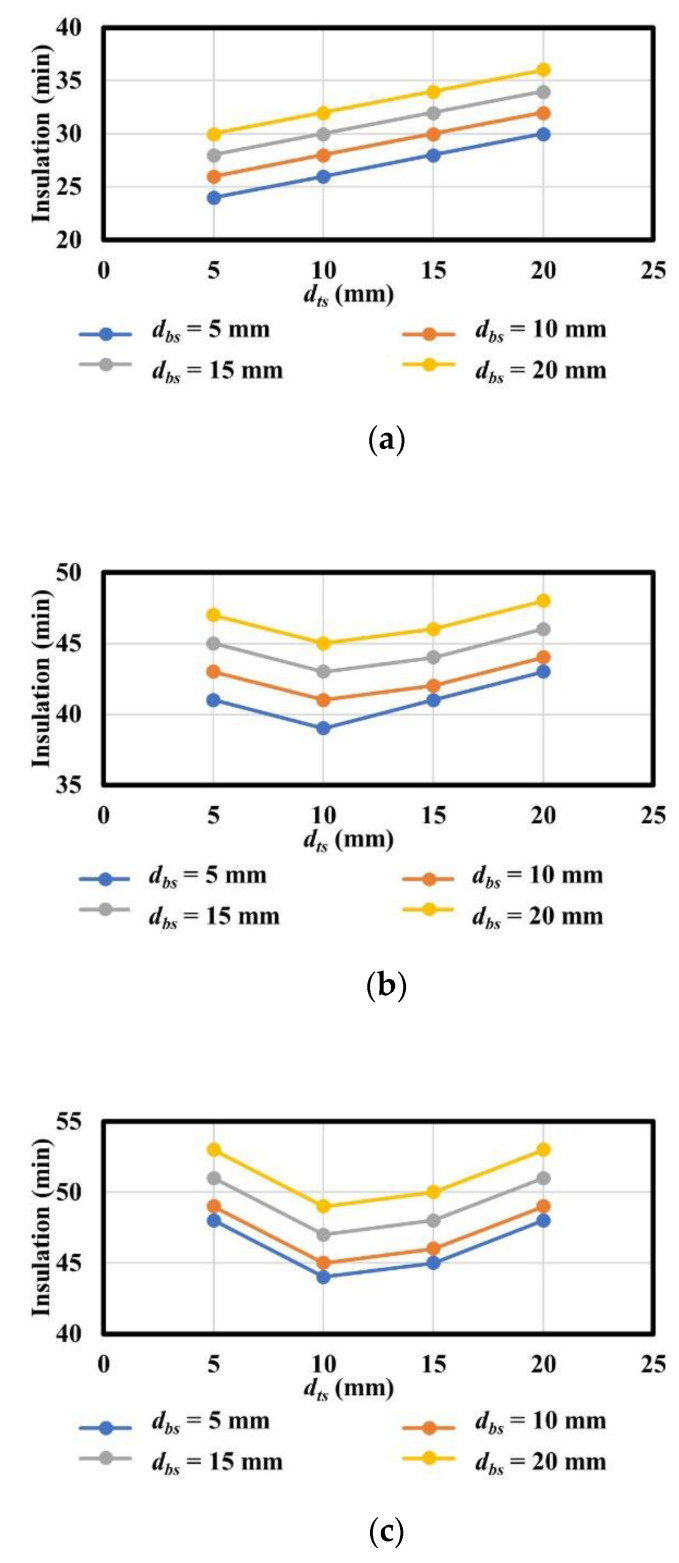
Summary of analysis results with constant *d_ts_*: (**a**) *d_p_* = 20 mm with varying *d_bs_*; (**b**) *d_p_* = 40 mm with varying *d_bs_*; (**c**) *d_p_* = 60 mm with varying *d_bs_*.

**Figure 20:**
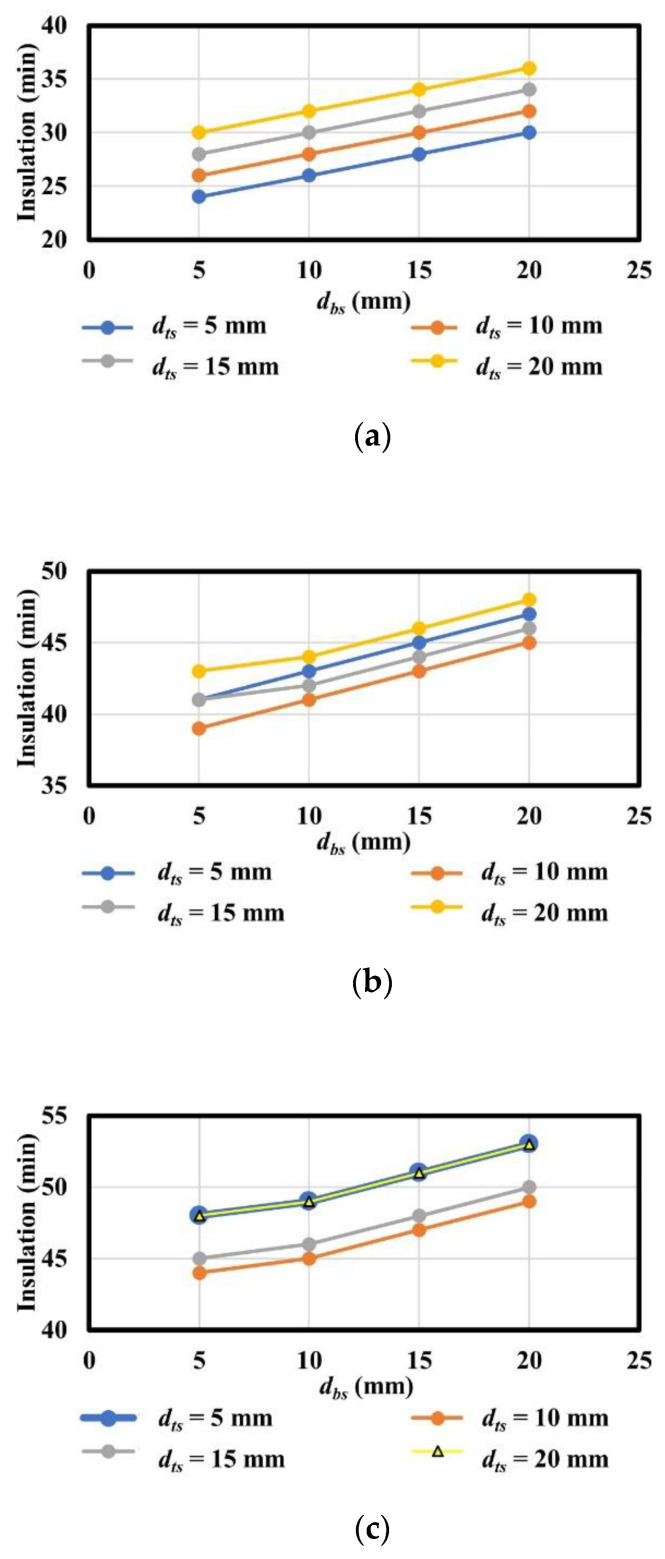
Summary of analysis results with constant *d_bs_*: (**a**) *d_p_* = 20 mm with varying *d_ts_*; (**b**) *d_p_* = 40 mm with varying *d_ts_*; (**c**) *d_p_* = 60 mm with varying *d_ts_*.

Figure 19 and Figure 20 present a summary of the thermal insulation of the analysis model with various variables classified by the thickness of polymers (*d_p_*) in the thermal field.

According to the analysis results, the relationship between various thicknesses of top steel plates with constant thickness of polymers and those of the bottom steel plates and insulation exhibited a non-linear correlation. This is because the heat energy (*Q_sp_*) of polymers and that of steel alone (*Q_s_*) influence the temperatures of the unexposed surface, as shown in Figure 12. The heat energy (*Q_sp_*) of polymers is determined not only by the thermal properties of polymers and steel, but also by the interfacial properties between polymers and steel, whereas the heat energy of steel alone (*Q_s_*) is exclusively determined by the thermal properties of steel. This means that *Q_sp_* is the heat energy that passes through an obstacle caused by interfacial properties between polymers and steel, whereas *Q_s_* passes through the steel elements alone. In contrast, the relationships between various thicknesses of bottom steel plates with constant thickness of polymers and those of bottom steel plates and insulation exhibited an approximate linear correlation. This occurred because the thickness of the bottom steel plates influences the heat energy before approaching the parameter bar and polymers. Based on the correlations and conservative approach, the insulation performance of steel–polymer composite floors can be expressed in terms of the thickness of steel and polymers in the range 5 mm ≤ *d_ts_* ≤ 20 mm, 20 mm ≤ *d_p_* ≤ 60 mm, and 5 mm ≤ *d_bs_* ≤ 20 mm. The equation for insulation performance (I), presented in terms of *d_ts_*, *d_p_*, and *d_bs_*, is expressed in Equation (4).

(4) $$I\;\left(\min\right)=\left\{ \begin{array}{c} 1)\;d_{ts}>10\;\{\begin{array}{c} 6.5d_{p}+0.4d_{s}-100\;\left(20\leq d_{p}<40\right)\\ 0.2d_{p}+0.4d_{s}+24\;\left(40\leq d_{p}\leq 60\right) \end{array}\\ 2)\;d_{ts}\leq 10\;\{\begin{array}{c} 8.7d_{p}+1.2d_{ts}+0.4d_{bs}-0.04d_{p}d_{ts}-154\;\left(20\leq d_{p}<40\right)\\ 0.5d_{p}+1.2d_{ts}+0.4d_{bs}-0.04d_{p}d_{ts}+21\;\left(40\leq d_{p}\leq 60\right) \end{array} \end{array}\right.$$

## 5. Conclusions

In this study, steel–polymer composite floors were introduced as floor systems for steel structures. To evaluate the fire resistance of the composite floors, thermal behavior and temperature distribution should be prioritized. Temperature distributions that can be obtained by thermal behavior determine the mechanical properties, which exhibit different values depending on the temperature. Consequently, before investigating structural behavior under fire, fire resistance in the thermal field (insulation performance) should be investigated and temperature distribution must be obtained. Based on evaluations of the fire resistance performance of steel–polymer composite floors in the thermal field, the following points summarize the results presented herein.

(1) A reliable thermal-contact conductance-based thermal behavior analytical model, which was used in a previous study to estimate the temperature distribution of steel–polymer composite floors, was applied to a full-scale fire test of specimens to predict temperatures on unexposed surfaces. By comparing the test and analysis results obtained by the thermal behavior analytical model, the proposed analytical model is validated for applying full-scale fire tests.
(2) Based on the analysis results with various variables, such as the thickness of top and bottom steel plates and polymers, a database was obtained for applying the finite element model to the investigation of fire resistance in the structural field. Evaluating the fire resistance performance in a thermal field is a prerequisite for determining the element temperatures that influence mechanical properties depending on elevated temperatures.
(3) By investigating the correlation between various variables and the fire resistance of the composite floors in the thermal field, the insulation performance can be presented using simple equations. The proposed equations are defined based on the thickness of the top and bottom steel plates and polymers. Using the specific ranges of 5 mm ≤ *d_ts_* ≤ 20 mm, 20 mm ≤ *d_p_* ≤ 60 mm, and 5 mm ≤ *d_bs_* ≤ 20 mm, the equations are written as follows:
  $$I\;\left(\min\right)=\left\{ \begin{array}{c} 1)\;d_{ts}>10\;\{\begin{array}{c} 6.5d_{p}+0.4d_{s}-100\;\left(20\leq d_{p}<40\right)\\ 0.2d_{p}+0.4d_{s}+24\;\left(40\leq d_{p}\leq 60\right) \end{array}\\ 2)\;d_{ts}\leq 10\;\{\begin{array}{c} 8.7d_{p}+1.2d_{ts}+0.4d_{bs}-0.04d_{p}d_{ts}-154\;\left(20\leq d_{p}<40\right)\\ 0.5d_{p}+1.2d_{ts}+0.4d_{bs}-0.04d_{p}d_{ts}+21\;\left(40\leq d_{p}\leq 60\right) \end{array} \end{array}\right.$$
